# The effects of nitrogen fertilization on N_2_O emissions from a rubber plantation

**DOI:** 10.1038/srep28230

**Published:** 2016-06-21

**Authors:** Wen-Jun Zhou, Hong-li Ji, Jing Zhu, Yi-Ping Zhang, Li-Qing Sha, Yun-Tong Liu, Xiang Zhang, Wei Zhao, Yu-xin Dong, Xiao-Long Bai, You-Xin Lin, Jun-Hui Zhang, Xun-Hua Zheng

**Affiliations:** 1 Key Laboratory of Tropical Forest Ecology, Xishuangbanna Tropical Botanical Garden, Chinese Academy of Sciences, Mengla, Yunnan 666303, China; 2 Xishuangbanna Station for Tropical Rain Forest Ecosystem Studies, Chinese Ecosystem Research Net, Mengla, Yunnan 666303, China; 3 University of Chinese Academy of Sciences, Beijing 100039, China; 4 Guangxi Normal University, Guilin 541004, China; 5 Institute of Applied Ecology, Chinese Academy of Sciences, 72 Wenhua Road, Shenyang 110016, China; 6 State Key Laboratory of Atmospheric Boundary Layer Physics and Atmospheric Chemistry, Institute of Atmospheric Physics, Chinese Academy of Sciences, Beijing 100029, China

## Abstract

To gain the effects of N fertilizer applications on N_*2*_O emissions and local climate change in fertilized rubber (*Hevea brasiliensi*s) plantations in the tropics, we measured N_*2*_O fluxes from fertilized (75 kg N ha^−1^ yr^−1^) and unfertilized rubber plantations at Xishuangbanna in southwest China over a 2-year period. The N_2_O emissions from the fertilized and unfertilized plots were 4.0 and 2.5 kg N ha^−1^ yr^−1^, respectively, and the N_2_O emission factor was 1.96%. Soil moisture, soil temperature, and the area weighted mean ammoniacal nitrogen (NH_4_^+^-N) content controlled the variations in N_2_O flux from the fertilized and unfertilized rubber plantations. NH_4_^+^-N did not influence temporal changes in N_2_O emissions from the trench, slope, or terrace plots, but controlled spatial variations in N_2_O emissions among the treatments. On a unit area basis, the 100-year carbon dioxide equivalence of the fertilized rubber plantation N_2_O offsets 5.8% and 31.5% of carbon sink of the rubber plantation and local tropical rainforest, respectively. When entire land area in Xishuangbanna is considered, N_2_O emissions from fertilized rubber plantations offset 17.1% of the tropical rainforest’s carbon sink. The results show that if tropical rainforests are converted to fertilized rubber plantations, regional N_2_O emissions may enhance local climate warming.

Nitrous oxide (N_2_O) is a major greenhouse gas that contributes to the destruction of the protective ozone layer in the stratosphere and to global warming^1^,^2^,^3^. Its global warming potential (GWP) is about 310 times that of carbon dioxide (CO_2_) on a 100-year time scale (i.e., the 100-year CO_2_ equivalence (100a-CO_2_ eq) of N_2_O)^2^,^3^,^4^,^5^. Nitrous oxide has been reported to account for between 2–4% and 44%^3^,^4^,^6^ of the total GWP of greenhouse gases. The wide range of these estimates highlights the great uncertainty surrounding regional N_2_O emissions and their GWP at the global scale. Hence, N_2_O fluxes and their contributions to GWP feedback at local and global scales require more attention.

In recent decades, as N fertilizer applications and N deposition have increased worldwide, global levels of N_2_O have increased, resulting in changes in the N cycle^1^,^2^,^5^,^6^,^7^,^8^. In tropical regions, large quantities of fertilizers are applied to both farmland and agroforestry systems^9^,^10^,^11^,^12^,^13^,^14^ to meet the demands for high agricultural production. Chronic N deposition will cause N_2_O emissions to increase from their present levels by several fold^12^,^13^,^14^,^15^,^16^. The magnitude of the increase in N_2_O emissions depends on the soil N contents, the physico-chemical^17^,^18^,^19^,^20^,^21^,^22^,^23^ and biomass^24^,^25^,^26^ characteristics of the soil, environmental factors, and substrate quality and quantity^2^.

On the global scale, tropical rainforest soils account for 14–23% of the atmospheric N_2_O budget^27^. For example, it has been estimated that soil emissions represent around 57% of the global atmospheric sources of N_2_O^28^. Soil fertilization is one of the major contributors to N_2_O emissions and accounts for more than 51% of the total emissions from plantation forests^29^. Elevated applications of synthetic N fertilizers, coupled with land use and climate changes, are the main factors that control N_2_O emissions from tropical soils. Wang *et al.*^16^ reported that NH_4_^+^-N fertilizer was responsible for higher soil N_2_O emissions than nitrate nitrogen (NO_3_^−^-N) fertilizer, as in their study the nitrification rates were higher from NH_4_^+^-N-treated plots than from NO_3_^−^-N-treated plots in subtropical forests. Few studies have examined changes in soil N_2_O emissions in response to the ongoing deposition of different forms of N in tropical forests in China. Hence, to support both estimations of the contributions of tropical forest soils to local and global N_2_O emission budgets and predictions of future trends in N_2_O emissions, it is important to quantify the N_2_O emissions from these forest soils in response to various N inputs. Therefore, the results of such a study will improve our understanding of the effects of chronic N inputs to tropical forest soils.

High global demand for natural rubber has resulted in sharp price increases. The unique climatic and growing conditions required by rubber trees mean that plantations are mainly concentrated in areas to the south of the Amazon River, western Peru, southern Bolivia and Brazil, and in some developing countries in Southeast Asia^30^. Rubber plantations in China have traditionally been planted in the south and southeast of the country. However, the ongoing expansion of rubber plantations has resulted in further loss of virgin tropical rainforest in other areas^31^,^32^,^33^,^34^. For example, in Xishuangbanna, southwest China, rubber plantations cover an area of ~4.7 × 10^5^ ha (24.6% of the total land area), which represents about half the tropical rainforest in the region^34^.

Intensive soil-management practices, along with chronic additions of synthetic N fertilizers, mean that rubber plantations are potential N_2_O sources in tropical regions. Furthermore, while high rainfall, soil moisture content, soil temperature, and soil nutrient availability provide favorable environmental conditions for microbial activities, they are also important environmental factors that regulate N_2_O emissions from tropical forest soils. The main objective of the present study was therefore to improve our understanding of how soil N_2_O emissions are influenced by nitrogen (N) fertilizers in tropical rubber plantations by evaluating N_2_O emissions from rubber plantations in Xishuangbanna. The specific aims are to (1) quantify the influence of synthetic N fertilizers on N_2_O emissions, (2) assess spatial and temporal variations in N_2_O fluxes, (3) identify the time and extent of the effect of mineral N fertilizer applications on N_2_O fluxes, and (4) identify the environmental factors that control N_2_O fluxes in tropical rubber plantations.

## Results

### Site conditions at the rubber plantation

**Figure 1:**
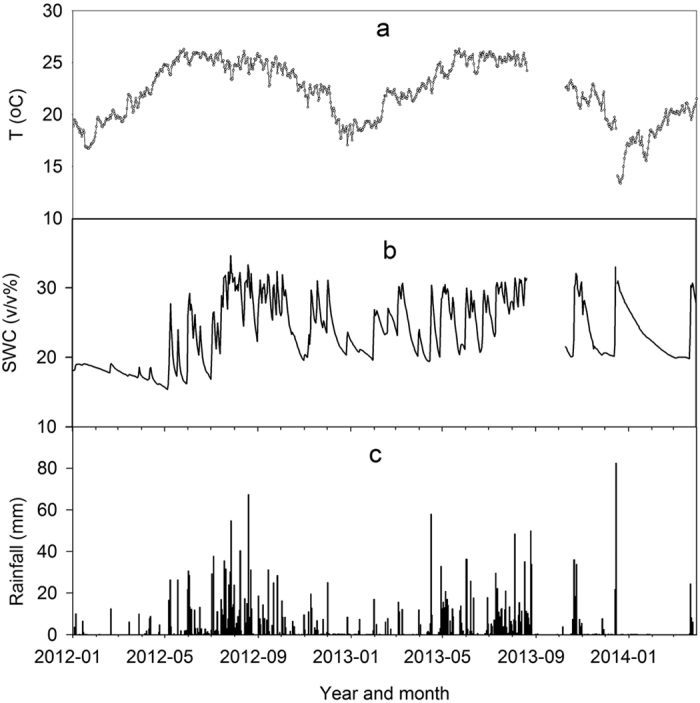
(**a**) Soil temperature at 5 cm depth, (**b**) soil water content (v/v%) at 0–10 cm depth, (**c**) and rainfall dynamics in the rubber plantation, Xishuangbanna, southwest China.

The results show that temporal variations in soil temperature (0–5 cm soil depth) followed a unimodal pattern, with the highest temperature recorded in the middle of the rainy season. The total rainfall from 1 April 2012 to 31 March 2013 was 1292.6 mm, and from 1 April 2013 to 31 March 2014 was 1187.3 mm; 80.0% of the rainfall and 83.6% of the rainfall events occurred during the rainy season. Only 13 rain events occurred during the dry season (Fig. 1a). The soil water content increased sharply because of high rainfall that occurred at the end of April after a relatively long dry period (Fig. 1b,c). There were strong positive correlations between soil temperature and soil water content in all the treatments and at the flux tower (Table S1). The temperature and soil water content measurements made at the flux tower, along with other parameters, were included in the correlation analyses.

**Figure 2:**
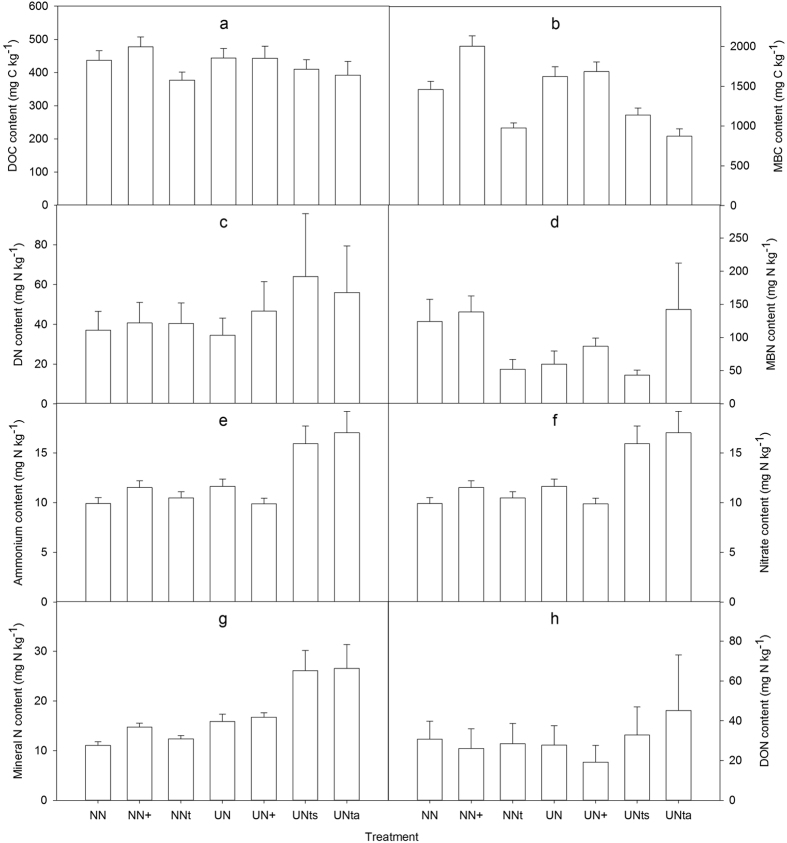
(**a**) Area-weighted value of soil temperature at a depth of 5 cm, (**b**) soil water content (v/v%) at 0–10 cm depth, (**c**) Dissloved organic carbon (DOC), (**d**) Microbe biomass carbon (MBC), (**e**) Dissolved nitrogen (DN), (**f**) Microbe biomass nitrogen (MBN), (**g**) NH_4_^+^-N, (**h**) NO_3_^−^-N, (**i**) mineral N, and (**j**) Dissolved organic nitrogen (DON) for the fertilized and unfertilized treatments in the rubber plantation in Xishuangbanna, southwest China.

The temporal variations in soil temperature (F = 0.316, p = 0.929) and soil water content (F = 0.368, p = 0.692) for the different treatments were not significant. However, we found significant differences in dissolved organic carbon (DOC) (F = 2.20, p = 0.037), microbial biomass carbon (MBC) (F = 32.34, p < 0.0001), NH_4_^+^-N (F = 6.38, p < 0.0001), NO_3_^−^-N (F = 4.12, p = 0.0002), and mineral N (F = 6.55, p < 0.0001) over the monitoring period (Fig. 2). There were no clear seasonal trends in C and N concentrations (Fig. S1). The DOC, MBC, dissolved nitrogen (DN), NH_4_^+^-N, and dissolved organic nitrogen (DON) were higher in the unfertilized plots than fertilized plots, whereas NO_3_^−^-N was higher in the fertilized plots than unferitilized plots (Table 1). This clearly indicates that the fertilizer had a considerable effect on soil chemical properties in the rubber plantations after 2 years of N fertilizer applications.

### Fertilizer effects on N_2_O emissions in the rubber plantation

**Figure 3:**
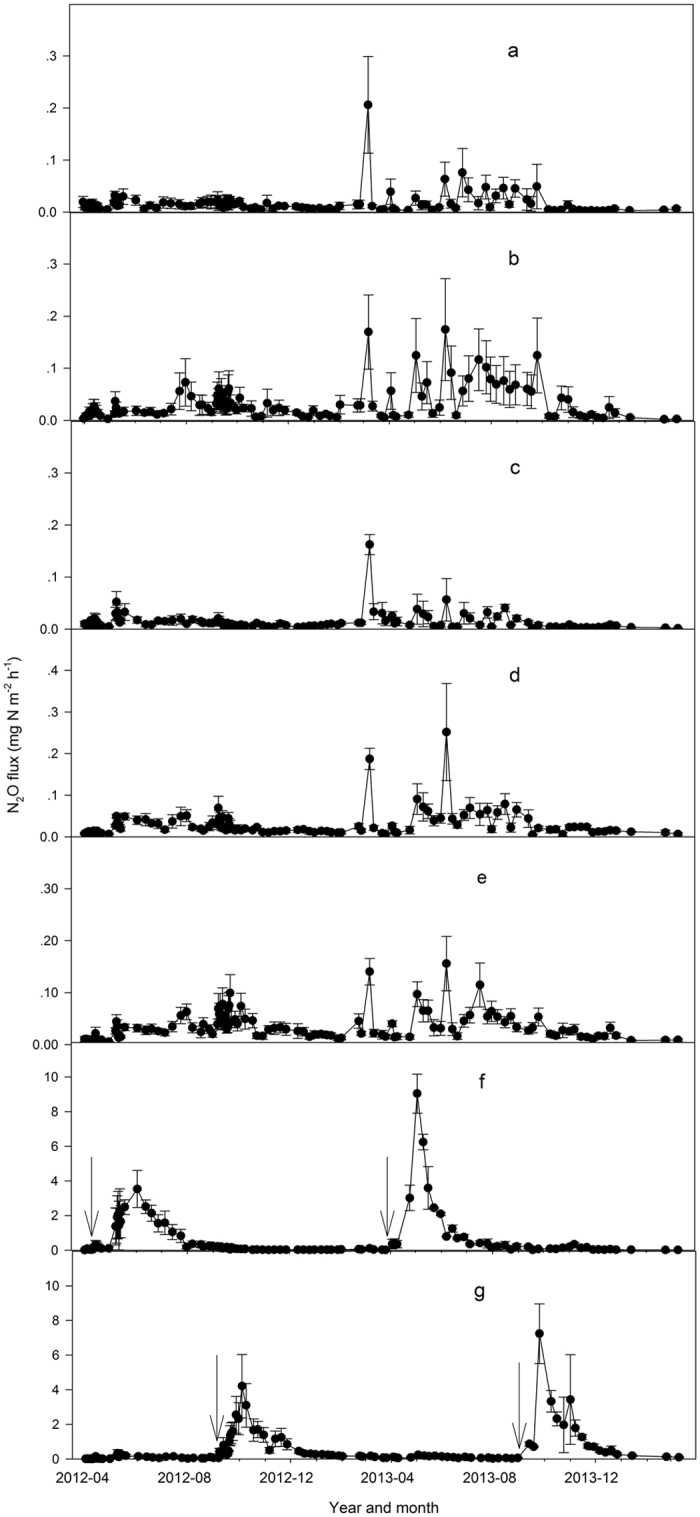
N_2_O fluxes from the fertilized treatments in the rubber plantation in Xishuangbanna, southwest China, showing data for the platform [NN, (**a**)], slopes [NN+, (**b**)], and old fertilizer trench [NNt, (**c**)] of the unfertilized treatment, and the platform [UN, (**d**)], slope [UN+, (**e**)], dry season fertilizer trench [UNts, (**f)**], and rainy season fertilizer trench [UNta, (**g**)] of the fertilized treatment. The arrows in (**f**,**g**) indicate the fertilizer date.

**Figure 4:**
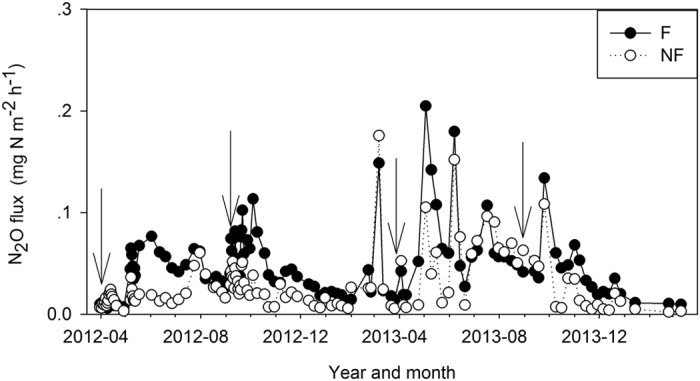
Area weighted mean N_2_O fluxes from the fertilized and unfertilized treatments during the observation period. A black circle indicates the area-weighted N_2_O flux from the fertilized treatment. An empty circle indicates the area weighted mean N_2_O flux from the unfertilized treatment. The arrow indicates the date of fertilization.

The N_2_O flux rates showed similar seasonal trends in the unfertilized platform plots (NN), unfertilized slope plots (NN+), unfertilized trench plots (NNt), fertilized platform plots (UN), and fertilized slope plots (UN+) (Fig. 3a–e), with high flux rates during the rainy season when the soil temperature, soil water contents, and rainfall were high, and low flux rates during the dry season, when the soil water contents and soil temperature were low (Fig. 1a,b). The trends in N_2_O fluxes at the dry season fertilizer trench plots (UNts) and rainy season fertilizer trench plots (UNta) were different from those from the unfertilized platform, unfertilized slope, unfertilized trench, fertilized platform, and fertilized slope treatments. A month after fertilizer application during the dry season, the N_2_O fluxes increased sharply to a maximum and remained at a relatively high level about 4 months until the end of August (Fig. 3f). Similarly, during the rainy season the N_2_O fluxes increased rapidly at ~15 days after fertilizer application and remained high for about 3 months (Fig. 3g). After fertilizer application, it took much less time to reach the peak N_2_O flux in the rainy season than in the dry season. Likewise, the peak N_2_O flux that occurred after fertilizer application was maintained for a longer duration (about 4 months) in the rainy season than in the dry season (about 3 months). Because the fertilizer was applied during April and September, the area-weighted N_2_O flux from the fertilized rubber plantation showed a bimodal pattern (Fig. 4).

The mean N_2_O flux rates varied significantly (F = 23.14, *p* < 0.0001) among the different treatments during the monitoring period (Fig. 3; Table S3). The flux rate was highest at the fertilized trench during the rainy season (0.58 ± 0.11 mg N m^−2 ^h^−1^), closely followed by the dry season fertilized trench (0.55 ± 0.092 mg N m^−2 ^h^−1^), and was lowest at the unfertilized trench (0.014 ± 0.0016 mg N m^−2 ^h^−1^). The area-weighted N_2_O flux rate was significantly higher (t = 8.1, p < 0.0001, df = 118) at the N fertilized plots (0.046 ± 0.032 mg N m^−2 ^h^−1^) than the unfertilized plots (0.029 ± 0.026 mg N m^−2 ^h^−1^). The N_2_O fluxes from both fertilized and unfertilized plots were higher during the rainy season than during the dry season. The annual cumulative N_2_O fluxes from the fertilized and unfertilized treatments were 4.0 and 2.5 kg N ha^−1^ yr^−1^, respectively. The N_2_O emission factor was 1.96% at the rubber plantation.

### Factors influencing N_2_O emissions at the rubber plantation

**Figure 5:**
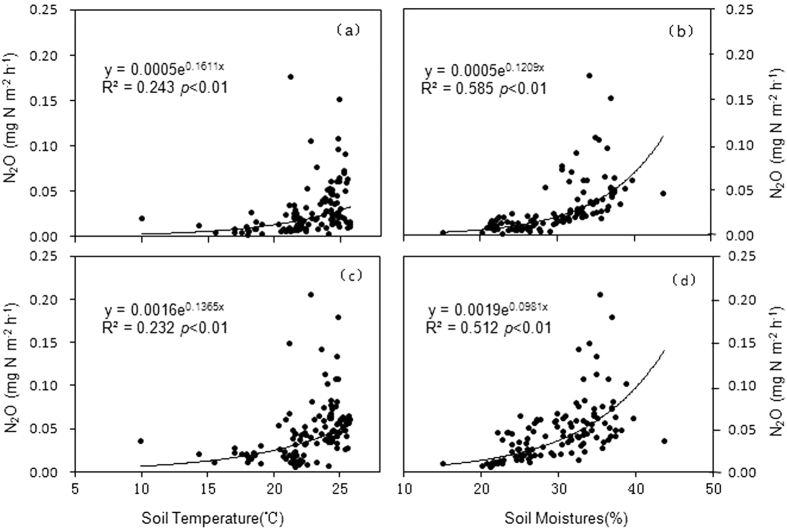
Regression equation between area weighted mean nitrous oxide emissions (mg N m^−2 ^h^−1^) and soil temperature (0–5 cm depth), and soil water content (0–10 cm) in the rubber plantation at Xishuangbanna, southwest China. (**a**) Soil temperature versus area weighted mean N_2_O from the unfertilized treatment, (**b**) soil water content versus area weighted mean N_2_O flux from the unfertilized treatment, (**c**) soil temperature versus area weighted mean N_2_O from the fertilized treatment, and (**d**) soil water content versus area weighted mean N_2_O flux from the fertilized treatment.

With the exception of the fertilizer trench in the rainy season, regression analysis shows significant correlations among soil temperature, soil water content, and N_2_O flux rates for the different treatments (Table S2). In contrast, there were no clear correlations between C fractions and the N_2_O flux rates or between the N fractions and the N_2_O flux rates (data not shown). The N_2_O flux rate was higher from the fertilized plots than from the unfertilized plots (Fig. 4), which reflects the fact that soil water content and temperature had a greater influence on the flux rates in unfertilized than did fertilized treatment, and that fertilizer applications decreased the influence of soil water content and soil temperarue on flux rates (Fig. 5). Regression analysis between soil temperature and N_2_O flux (Fig. 5) shows that Q10 dependence on the temperature of the unfertilized treatment (5.0) was higher than that of the fertilized treatment (3.9). Furthermore, Pearson correlations show that of the different C and N fractions, NH_4_^+^-N was significantly and positively correlated with the N_2_O flux rate from the fertilized (r = 0.45, p = 0.0063, n = 36) and unfertilized treatments (r = 0.37, p = 0.030, n = 36).

We used Pearson correlation analysis to assess the relationships among N_2_O flux, C and N fractions, and SWC in the different treatments. The results indicate that the N fractions, such as DN, DON, mineral N, NH_4_^+^-N, and NO_3_^−^-N, were significantly (*p* < 0.01) and positively correlated with the average N_2_O fluxes (Table 3). Stepwise linear regression analysis also indicated that the NH_4_^+^-N content was the key control on variations in the N_2_O fluxes from the different treatments. The regression equation was as follows:


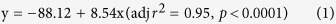


where y is the annual N_2_O flux (kg N ha^−1^ yr^−1^) and x is the average NH_4_^+^-N content (mg N kg^−1^).

## Discussion

**Figure 6:**
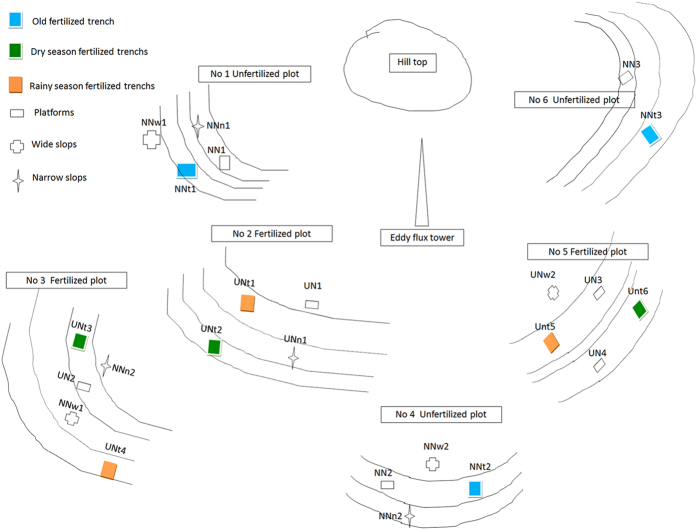
Experimental design of the study in a rubber plantation in Xishuangbanna, southwest China. Blue rectangles indicate the old fertilized trench (NNt) in the unfertilized treatment. Green rectangles indicate the dry season fertilized trench (Unts) in the fertilized treatment. Red rectangles indicate the rainy season fertilized trench (Unta) in the fertilized treatment. Empty rectangles indicate the platform (NN) of unfertilized and fertilized (UN) treatments, respectively. Crosses indicate the wide slopes of the unfertilized (NNw) and fertilized (UNw) treatments, respectively. Stars indicate narrow slopes of the unfertilized (NNn) and fertilized treatments (UNn), respectively.

Nitrogen fertilization has a significant influence on N_2_O dynamics and contributes to high N_2_O fluxes^5^,^35^,^36^. The results from this study show that the N_2_O flux was higher from the fertilized rubber plantation (4.0 kg N ha^−1^ yr^−1^) than from the unfertilized rubber plantation (2.5 kg N ha^−1^ yr^−1^). The increased fertilizer ratio (160%) of the rubber plantation was less than the ratios reported for subtropical forests in southeast China (403–762%), where the fertilizer application rates ranged from 40 to 120 kg N ha^−1^ yr^−1^ 16. Although the amount of N fertilizer (75 kg ha^−1^ yr^−1^) applied to the rubber plantation was higher than that reported in some previous studies^10^,^16^,^37^,^38^, which showed the contribution of inorganic N fertilizer applications to N_2_O emissions in the rubber plantation at Xishuangbanna were much lower. This result can be explained as follows. Firstly, fertilization in the rubber plantation followed the furrow fertilization model (Fig. 6). This meant that the fertilized trench plot was a hot spot of N_2_O emissions from which the emissions were significantly higher than from the slope and terrace plots (Fig. 3; Table S3). The fertilized trench plots, however, accounted for only a small areal proportion (Fig. 6) of the rubber plantation, therefore the overall contribution of the fertilized trench plots on N_2_O emissions were low. Secondly, there were no significant (p > 0.05) differences among the N_2_O fluxes from the fertilized and unfertilized slope and terrace plots (Fig. 3; Table S3). Hence, the area-weighted mean N_2_O flux from the fertilized plot was only 1.6 times greater than that from the unfertilized plot.

As increased amounts of fertilizer are applied, N_2_O emissions increase^39^,^40^,^41^. The N_2_O emissions from the fertilized rubber plantation (N application rate was 75 kg ha^−1^ yr^−1^) of the present study were lower than those from plantations where the N fertilizer application rates were higher (130–360 kg N ha^−1^ yr^−1^), such as papaya orchards (3.8–5.4 kg N ha^−1^ yr^−1^), coffee plantations (4.3 kg N ha^−1 ^yr^−1^), coffee + tree legumes in Costa Rica (5.8 kg N ha^−1 ^yr^−1^), and lychee orchards (4.5 ± 1.1 kg N ha^−1 ^yr^−1^)^39^,^40^,^41^. The fertilization emission factor (1.96%) reported here was higher than those reported for the lychee (1.56%)^39^, papaya (0.90%)^41^, coffee+tree legume orchards (~1.0%)^40^ in tropical regions, and the default factor (1.0%) reported by the IPCC^5^. This shows that the N_2_O emission ratios for N-fertilized rubber plantations were higher than for other plantation types, which may have significant implications for the local GWP.

Differences in N_2_O emissions among treatments in the rubber plantation can be attributed to the combined effects of environmental, soil, vegetation, and human factors. The combined effects can be explained by soil physical and biochemical characteristics that contributed directly to N_2_O emissions. The significant positive correlations between the N_2_O flux and the contents of organic N fractions, and between the N_2_O flux and mineral N fractions, indicate that N was the most important control on spatial variations in N_2_O emissions (Table 3). Furthermore, stepwise linear regression showed that the NH_4_^+^-N content explained 94.5% of the variance in N_2_O among the treatments, and that the NH_4_^+^-N fraction dominated the spatial variations among the treatments. After 2 years without fertilizer application, the microbiomass community, which did not show the same degree of variation in the MBC and MBN (Fig. 2b,d)^21^, may vary with changes in the NH_4_^+^-N content, resulting in changes in N_2_O emissions from different treatments in the rubber plantation examined in the present study.[Table t2]

The NH_4_^+^-N content, along with other factors, contributed to spatial variations in the N_2_O emissions from the different treatments. It was also one of the main controls on the area weighted mean values of N_2_O in the fertilized and unfertilized rubber plantation. This suggests that nitrification rather than denitrification controlled N_2_O emissions in the rubber plantation. It also contrasts with results from other studies in rubber plantation sites that are controlled by nitrification^10^,^42^,^43^, in which N_2_O emissions have generally been attributed to the combined effects of environmental, soil, vegetation, and human factors. While the soil pH in the rubber plantation of the present study (Table S4) was similar to that reported for other studies in tropical regions^12^,^44^,^45^, the water content of the filled pore space was less than 80% (Fig. S2), which triggered changes in the threshold of the soil microbiomass community for denitrication^21^,^22^,^23^. Thus, NH4^+^-N and N_2_O emissions were significantly and positively correlated in the present study predicted nitrification may the main N_2_O emission way in this rubber plantation. To understand the influence of the previously mentioned factors on N_2_O emissions, further studies are needed to (1) determine whether nitrification is the main control on N_2_O emissions in each treatment, (2) calculate the N_2_O:nitrification ratio in the rubber plantation, and (3) examine the N_2_O emission processes in isotope incubation experiments, in the field and the laboratory.

Regardless of whether nitrification or denitrification dominates, the soil water content and temperature are the most important controls on N_2_O seasonal dynamics, and drive higher N_2_O emissions in the rainy season than in the dry season in most forests. The area-weighted mean N_2_O fluxes from each treatment indicate that the N_2_O fluxes were higher in the rainy season than in the dry season (Figs 2 and 4). With the exception of the fertilizer trench plot during the rainy season, the seasonality in the N_2_O flux rate can be explained by the significant correlations between N_2_O flux and soil temperature, and between N_2_O flux and soil water content (Figs 3 and 5; Table S1). Seasonal variations in N_2_O fluxes from the rainy season fertilizer trench plot were not well correlated with soil temperature, because the flux rate increased gradually after the rainy season fertilizer application as the soil temperature decreased (Figs 1a and 3g). Thus, the timing of fertilizer applications may also have a significant influence on the relationship between soil temperature and N_2_O fluxes^35^,^36^,^39^, and may cause variations in N_2_O flux rates within a given plantation. The N_2_O flux reached a peak more quickly after fertilization in the rainy season fertilizer trench plot than in the dry season fertilizer trench plot (Fig. 3f,g). Because of the relatively high soil water content and temperature in the later part of the rainy season (Fig. 1b), there was more microbiomass to produce N_2_O emissions^24^,^26^, so the increased N mineralization rate and N_2_O flux were sustained for a shorter time in the rainy season than after fertilization in the dry season^39^. In addition, fertilizer application reduced the sensitivity of N_2_O emissions to temperature and moisture fluctuations because of variations in the N input to the substrate (Fig. 5; Fig. S1). The fact that N_2_O was more responsive to variations in soil moisture than soil temperature indicates that N cycling processes are relatively sensitive to variations in soil moisture because of the relatively small variations in temperature in the rubber plantation^24^,^25^,^40^,^42^.

The soil C and N fractions are the major energy sources for microbial activity and the substrate, respectively, which produce N_2_O emissions in tropical forests soil^42^,^43^,^44^,^45^,^46^. However, in this study there were no clear correlations between N_2_O fluxes and any of the different soil N or C fractions for any of the treatments. In addition, transfers in the microbiomass community via soil water may not be clear from the MBC and MBN dynamics^24^,^25^,^26^ and, based on our results, the MBC and MBN for each treatment are not correlated to N_2_O fluxes. Thus, variations in the N and C fractions had little influence on temporal variations in N_2_O fluxes in all the treatments. Furthermore, the lack of clear trends in the contents of the C and N fractions in all the treatments (Fig. S1) may mean that a combination of other factors influenced N_2_O emissions. Temperature, soil moisture, and microbial bioactivity stimulate the N cycle^14^,^42^,^44^. Variations in N uptake from the surface and interflow runoff discharges of N influence the supply of N_2_O emissions to the substrate^13^,^45^,^46^,^47^. Rubber tree phenology and changes in fine root dynamics in the rhizosphere microenvironment influence N and C availability^47^,^48^,^49^. Furthermore, variations in litter inputs and soil microbial biomass influence the soil C and N fractions^39^,^44^,^45^,^46^,^47^, and, depending on the substrate and the energy supply, fertilization will have a considerable influence on N_2_O emissions^33^,^43^,^44^.

Overall, N_2_O from the fertilized rubber plantation contributes about 1227.6 kg C ha^−1 ^yr^−1^ to the 100-year GWP, which accounts for 6.0% of the rubber plantation net ecosystem exchange (NEE) (9.04 t C ha^−1 ^yr^−1^)^49^. This figure is far less than that reported for an acacia plantation in Sumatra, Indonesia (10.0%)^37^, and shows that the N_2_O fluxes from the rubber plantation made a relatively small, yet significant, contribution to the GWP^17^. However, the NEE is similar to that reported for a Chinese chestnut plantation, where CO_2_ emissions accounted for more than 95.0% of the total GWP, regardless of the understory management treatment^50^. Comparison with the results from the unfertilized rubber plantation showed that mineral fertilizer only contributed 1.5 kg N ha^−1 ^yr^−1^ or a GWP of 708.8 kg CO_2_ ha^−1 ^yr^−1^, which offset the NEE of the rubber plantation by 2.1%. A modeling study^7^ has shown that the relatively low contribution of N_2_O to the GWP in the rubber plantation can be attributed to increases in the C sink potential and reductions in the GWP arising from the use of N fertilizer^17^,^50^,^51^.

In this study, the ratio of the rubber plantation N_2_O to the primary tropical forest NEE was estimated at 1.67 t C ha^−1^ yr^−1^ 52 (Table 3). The ratio of the N_2_O GWP of the fertilized rubber plantation to the primary tropical rainforest NEE was relatively high (31.5%), and was 8.6 times greater than the N_2_O GWP:NEE ratio of the tropical rainforest. Most of the conversion from tropical rainforest to rubber plantation in Xishuangbanna has occurred since the 1970s^31^,^32^,^33^,^34^. In Xishuangbanna during 2014, rubber plantation and tropical rainforest covered areas of 47.12 × 10^4^ hm^2^ and 86.83 × 10^4^ hm^2^, respectively^34^. Using the land use ratio (i.e., the ratio of the fertilized rubber plantation N_2_O GWP to the primary tropical forest NEE), we calculated that for the GWP the rubber plantation will offset 17.1% of the NEE of the primary tropical rainforest in Xishuangbanna. This indicates that as the area of the rubber plantation increases, the local tropical rainforest carbon sink will decrease because of the contribution of fertilizer in the rubber plantation, and there will be positive greenhouse effect feedbacks to the local climate with particular influences on precipitation and temperature^53^. Greenhouse gas emissions change with variations in land use and vegetation life cycles^1^,^2^,^5^,^6^,^8^. In the present study, we investigated only one stage of the life cycle of a mature rubber plantation. Hence, to obtain more accurate information about N_2_O feedback from fertilized rubber plantations to the local GWP, and the total accumulated greenhouse gas feedback to local climate change, future studies should investigate the whole life cycle of the rubber plantation.

## Materials and Methods

### Site description

The study site was a managed rubber plantation (21°55′30″N, 101°15′59″E; elevation 580 m) that was planted in 1993 in Xishuangbanna, Yunnan Province, China. The annual average temperature in this area is 21.7 °C and the mean annual precipitation is 1557 mm^53^. There is a rainy season from May to October and a dry season from November to March; the dry season is further divided into a cool, dry period from November to February, and a hot, dry period during March and April^54^. The tropical rainforest was felled in 1990, and rubber trees were planted in May 1993. Rubber trees were planted 2 m apart in rows. The spacing between adjacent rows was either narrow (3 m) or wide (19 m), and the trees were planted at a density of 495 rubber trees per hectare. The mean canopy height of the rubber plantation area ranged from 20 to 30 m. The main rubber-tapping period is from May to November. The plantation was not irrigated. Fertilizer was applied twice each year, during the dry season at the end of March (or the beginning of April) and during the rainy season at the end of August or the beginning of September. The study site is on a slope (15%) and is covered with a thick layer of oxisol, the physical and chemical characteristics of which are listed in Table S4.

### Experimental design

In March 2012 we established a 1 ha study plot in the rubber plantation. We set up a randomized block experiment to compare the effects of mineral fertilizers on N_2_O emissions from the terraces, slope, and fertilizer trench. Taking into account the slope gradient, slope direction, and buffer zone, three fertilized and unfertilized blocks were installed from the bottom to the top of the slope around an eddy meteorological observation tower (on top of the hill) (Fig. 6). There were three terraces (NN), three fertilizer trenches (NNt), two narrow slopes, and two wide slopes (NN+) in the unfertilized plots, and four terraces (UN), three dry season fertilizer trenches (UNts), three rainy season fertilizer trenches (UNta), two narrow slopes, and two wide slopes (UN+) in the fertilized plots. In line with local farming practices, 1 kg of mineral fertilizer (Hubei Sanning Chemical, China) containing 15% N as (NH_2_)_2_CO, 15% P as NH_4_H_2_PO_4_, and 15% K as KCl was applied to each rubber tree every year; this amounted to an application rate of 75 kg N ha^−1^ yr^−1^, split into two applications (1 April and 6 September in 2012 and 2013). The fertilizer was applied by hand into a trench that was 150 cm long, 20 cm wide, and 10 cm deep between two rubber trees at a rate of 0.5 kg per tree per application.

### Measurement of N_2_O emissions

We measured the N_2_O fluxes simultaneously in all the fertilized and unfertilized treatments for 2 years from 1 April 2012 to 30 March 2014 using a static opaque chamber technique^20^,^41^,^52^,^53^. A chamber base collar made of PVC casing (covering an area of 0.12 m^2^) was inserted to a depth of 0.10 m in the center of each field plot to measure the gas flux. The chamber base collars were kept in place throughout the entire measurement period. Chambers, 0.12 m^2^ in area and 0.2 m high, were manually mounted onto the base collars to measure gas fluxes. To measure the N_2_O flux, five gas samples were taken from each chamber during the 1 h incubation at 15 min intervals. The gas samples were collected and stored in 100 ml plastic syringes and then transported to the laboratory within 3 hours of sampling. All the gas samples were analyzed immediately in the laboratory using a gas chromatograph (GC, Agilent 7890A, Agilent Technologies, CA, USA) equipped with an electron capture detector at 330 °C following the DN–Ascarite method with N_2_ as the carrier gas and an ascarite filter to remove CO_2_ from the samples^20^,^51^,^55^,^56^. The N_2_O samples were isothermally separated in two stainless steel columns (both with an inner diameter of 2 mm, and either 1 or 2 m long) packed with Porapak Q, 80–100 mesh, at 55 °C. To check the accuracy of the sample analysis, four standard N_2_O samples (National Center for Standard Matters, Beijing, China) with concentrations of 350 ppbv were analyzed by the GC system every 10 samples. Results of the GC analyses were accepted when the four gas standard calibrations produced variation coefficients that were lower than 1%.

The air pressure and headspace air temperature were recorded when the samples were collected from the chamber enclosure. The gas flux was calculated from the change in concentration within the enclosure, the recorded air temperature, and pressure. We used either a linear or nonlinear fitting approach to determine the initial rate of change in the concentration and the N_2_O flux^55^,^56^,^57^. Sampling and measurements were carried out weekly throughout the observation period. All samples were collected between 09:00 and 11:00 AM (local time) on the sampling day so that the flux rate was representative of the mean daily flux^51^. Emissions for days lacking measurements were estimated as the arithmetic mean flux of the two closest days for which measurements were available. The daily estimates were summed to obtain the total weekly, monthly, and annual emissions.

### Auxiliary measurements

#### Soil volumetric water content and soil temperature

During gas sampling, the soil volumetric water content (0–12 cm soil depth) was determined with time-domain reflectometry (TDR100, Campbell Scientific, USA) in the soil close to the gas sampling plot. At the same time, the soil temperature (0–10 cm) and the air temperature were recorded with a needle thermometer. Missing soil water content and temperature data were calculated by calibrating the collected data with the soil water content (0–5 cm) and temperature, which was recorded half-hourly with a data logger fixed to the eddy flux tower (Campbell Scientific). Rainfall was recorded half-hourly by an automatic recorder attached to the top of the eddy flux tower.

#### Soil sampling and C/N fraction analysis

Throughout the soil N_2_O flux observation period, soil samples (~200 g) were collected at a depth of 0–20 cm every month from a point close to the static chambers using a stainless steel auger (3 cm in diameter). Once collected, soils were immediately passed through a 2 mm sieve to remove roots, gravel, and stones. All the soil analyses were completed within 48 hours. The soil samples were extracted in a 2.0 mol L^−1^ KCL solution (soil:water ratio of 1:10) and shaken for 1 h (160 rpm). The soil suspension was then filtered before NH_4_^+^-N and NO_3_^−^-N concentrations were determined with a continuous flow autoanalyzer (AutoAnalyzer 3; Germany). At the same time, a portion of each of the treated soil samples was pooled to make a composite sample for analysis of microbial biomass nitrogen (MBN), microbial biomass carbon (MBC), dissolved organic carbon (DOC), and total dissolved nitrogen (TDN). Soil MBC and MBN were determined by the chloroform fumigation–extraction method^58^. Four replicate samples of 7.0 g of each treated soil sample were fumigated with ethanol-free chloroform for 24 h at 25 °C in a sealed incubator in the dark. Three 7.0 g samples of each treated soil were not fumigated. After fumigation, the chloroform was completely removed from the soils, and both the fumigated and unfumigated samples were extracted with 35 ml of freshly prepared 0.05 M K_2_SO_4_ (soil:water ratio of 1:5), capped, and shaken at 300 rpm for 1 h. The suspensions were then centrifuged for 10 min at 5000 × g and the supernatants were filtered through 0.45 μm nitrocellulose membrane filters (Pall Life Science Company, Beijing, China). These filtered samples were analyzed for DOC and DN concentrations using Pt-catalyzed high-temperature combustion (680 °C) and a total organic carbon/total nitrogen analyzer (LiquiTOC II, Elementar Analyzer System, Germany). DOC and TDN were determined on the unfumigated filters, and the differences in DOC and TDN between the unfumigated and fumigated filters were the MBC and MBN, respectively. Mineral N was the sum of NH_4_^+^-N and NO_3_^−^-N, and DON was the difference between TDN and mineral N.

### Statistical analyses

The N_2_O flux from the fertilized and unfertilized areas was calculated as the area-weighted mean of three or more replicates from each treatment, as follows:


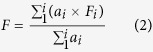


where *F* is the N_2_O flux (μg m^−2^ h^−1^), *a*_*i*_ is the treatment area (ha), *F*_*i*_ is the treatment N_2_O flux (μg m^−2^ h^−1^), and *i* is the treatment.

SPSS (version 16.0) software was used for the statistical analysis. One-way ANOVA and general linear models were used to examine differences in N_2_O, soil temperature, soil water content, DOC, MBC, DN, MBN, NH_4_^+^-N, NO_3_^−^-N, mineral N, and DON among the treatments. The Pearson correlation (two-tailed) test was used to determine the correlations between N_2_O and each of the environmental variables, and the soil C and N fractions. Stepwise linear regression analysis was performed with the N_2_O flux rate as the main factor. A paired *t*-test was used to detect differences among the soil temperature values, and among the area-weighted concentrations of N_2_O, soil water content, DOC, MBC, DN, MBN, NH_4_^+^-N, NO_3_^−^-N, mineral N, and DON for the fertilized and unfertilized treatments. All the data for N_2_O, soil temperature, soil water content, DOC, MBC, DN, MBN, NH_4_^+^-N, NO_3_^−^-N, mineral N, and DON, the different treatments, and the weighted area values were tested for normality with the Kolmogorov–Smirnov test (one sample), and all of the data complied with the assumptions of the analysis methods that we used. The N_2_O emission factor (EF) for rubber plantation soils after fertilizer had been applied was calculated as follows:


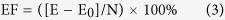


where E and E_0_ are the N_2_O emissions from the fertilized and unfertilized fields during the same period, respectively, and N is the amount of nitrogen fertilizer applied^9^.

## Additional Information

**How to cite this article**: Zhou, W.-J. *et al.* The effects of nitrogen fertilization on N_2_O emissions from a rubber plantation. *Sci. Rep.* **6**, 28230; doi: 10.1038/srep28230 (2016).

## Supplementary Material

Supplementary Information

## Figures and Tables

**Table 1:** Area weighted mean values (mean ± SE) and paired *t*-test results for DOC, MBC, DN, MBN, NH_4_^+^-N, NO_3_^−^-N, mineral N, and DON between the unfertilized and fertilized treatments in the rubber plantation.

Parameter	Unfertilized	Fertilized	t	p	df
DOC	578.6 ± 18.2	465.5 ± 26.1	5.24	<0.0001	18
MBC	2202.3 ± 82.3	1744.0 ± 80.7	7.18	<0.0001	18
DN	46.5 ± 11.9	35.7 ± 9.2	3.03	0.01	18
MBN	143.9 ± 26.1	80.7 ± 11.7	3.05	0.01	18
NH_4_^+^-N	13.5 ± 0.8	10.4 ± 0.6	7.50	<0.0001	36
NO_3_^−^-N	3.2 ± 0.5	6.5 ± 0.8	5.38	<0.0001	36
Mineral N	16.6 ± 0.9	16.8 ± 1.0	0.27	0.79	35
DON	30.6 ± 11.2	20.7 ± 8.8	2.79	0.01	18

**Table 2 t2:** Pearson’s correlations between the average N_2_O flux, soil water content, and fractions of C and N after the different treatments in the rubber plantation.

	SWC	DOC	DN	MBC	NH_4_^+^	NO_3_^−^	Mineral N	DON
r	−0.43	−0.53	0.89**	−0.69	0.98**	0.90**	0.80*	0.84**
p	0.29	0.19	0.003	0.06	<0.0001	0.003	0.017	0.009

^**^Correlation is significant at the 0.01 level (two-tailed).

^*^Correlation is significant at the 0.05 level (two-tailed).

n = 7.

**Table 3:** Global warming potential of N_2_O in the rubber plantation.

Treatments	F	NF	F-NF
N_2_O flux (kg N ha^−1 ^yr^−1^)	4.0	2.5	1.5
GWP	1929.1	1217.9	708.8
Ratio to the rubber plantation NEE (%)	5.8	3.7	2.1
Ratio to the tropical rainforest NEE (%)	31.5	19.9	11.6

F indicates fertilized rubber plantation; NF indicates unfertilized rubber plantation; F-NF indicates the difference between fertilized and unfertilized rubber plantation; TRF indicates tropical rainforest.

Rubber plantation and tropical rainforest NEE values were 9.04 t C ha^−1 ^yr^−1^ 49 and 1.67 t C ha^−1 ^yr^−1^ 52, respectively.

The global warming potential of N_2_O is 310 times that of CO_2_ over a period of 100 years^4^,^5^.
