# Supplementary material for: The effects of nitrogen fertilization on N2O emissions from a rubber plantation
Source: Sci Rep. 2016 Jun 21;6:28230. doi: 10.1038/srep28230 (PMC4915005; doi:10.1038/srep28230)
Supplement: Supplementary Information [file srep28230-s1.doc]

# The effects of nitrogen fertilization on N_2_O emissions from a rubber plantation

Wen-Jun Zhou ^1,2,3,+^ Hong-li Ji ^1,2,+^, Jing Zhu ^4^, Yi-Ping Zhang ^1,2^,*, Li-Qing Sha ^1,2^, Yun-Tong Liu ^1,2^, Xiang Zhang ^1,2^, Wei Zhao ^1,2,3^, Yu-xin Dong ^1,2,3^, Xiao-Long Bai ^1,2,3^, You-Xin Lin ^1,2,3^, Jun-Hui Zhang ^5^, Xun-Hua Zheng ^3,6^

1. Key Laboratory of Tropical Forest Ecology, Xishuangbanna Tropical Botanical Garden, Chinese Academy of Sciences, Mengla, Yunnan 666303, China
2. Xishuangbanna Station for Tropical Rain Forest Ecosystem Studies, Chinese Ecosystem Research Net, Mengla, Yunnan 666303, China
3. University of Chinese Academy of Sciences, Beijing 100039, China
4. Guangxi Normal University, Guilin 541004, China
5. Institute of Applied Ecology, Chinese Academy of Sciences, 72 Wenhua Road, Shenyang 110016, China
6. State Key Laboratory of Atmospheric Boundary Layer Physics and Atmospheric Chemistry, Institute of Atmospheric Physics, Chinese Academy of Sciences, Beijing 100029, China

*Corresponding author: Yi-Ping Zhang, email: [yipingzh@xtbg.ac.cn](mailto:yipingzh@xtbg.ac.cn), Tel: +86-871-5160904, Fax: +86-871-5160916

^+^ These authors contributed equally to this work.

**Fig. S1 (A, a)** Soil dissolved organic carbon (DOC), (B, b) Microbial biomass carbon (MBC), (C, c) dissolved nitrogen (DN), (D, d) Microbial biomass nitrogen (MBN), (E, e) ammonium, (F, f) nitrate, (G, g) mineral N, and (H, h) dissolved organic nitrogen (DON) content during the observation period in the rubber plantation in Xishuangbanna, Southwest China.

A indicates changes in the DOC contents of the unfertilized treatments; a indicates changes in the DOC contents of the fertilized treatments; B indicates changes in the MBC of the un fertilized treatments; b indicates changes in the MBC of the fertilized treatments; C indicates changes in DN fluxes from the unfertilized treatments; c indicates changes in DN fluxes from the fertilized treatments; D indicates changes in the MBN dynamics from the unfertilized treatments; d indicates changes in the MBN dynamics from the fertilized treatments; E indicates changes in the ammonium nitrogen content of the unfertilized treatments; e indicates changes in the ammonium nitrogen content of the fertilized treatments; F indicates the nitrate nitrogen content of the unfertilized treatments; f indicates the nitrate nitrogen content of the fertilized treatments; G indicates the mineral nitrogen (ammonium+ nitrate) content of the unfertilized treatments; g indicates the mineral nitrogen (ammonium+ nitrate) content of the fertilized treatments; H indicates the DON content of the unfertilized treatments; h indicates the mineral nitrogen DON content of the fertilized treatments.

**Fig.S2** Half-hour soil water filled pore-space dynamics in the rubber plantation.


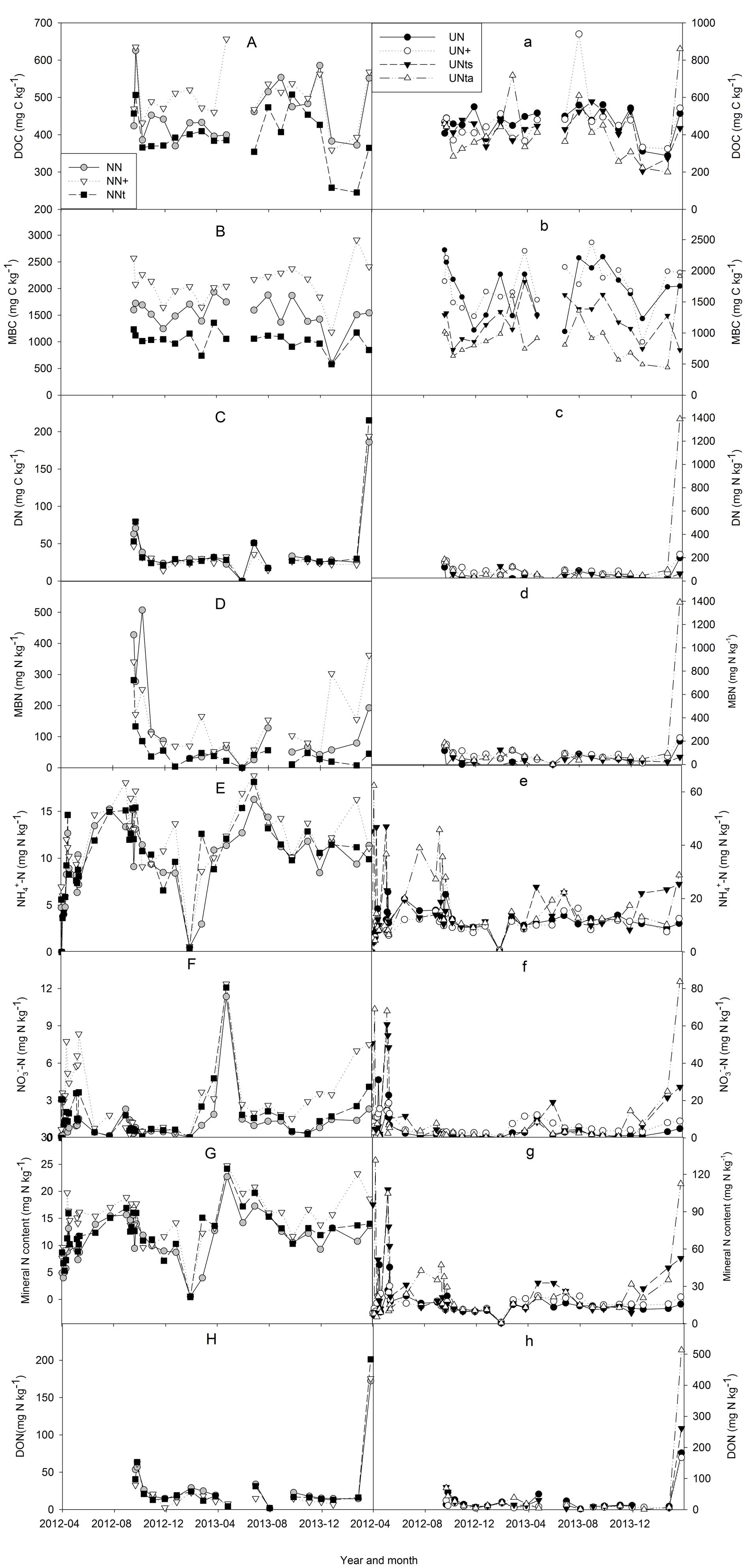
Fig. S1


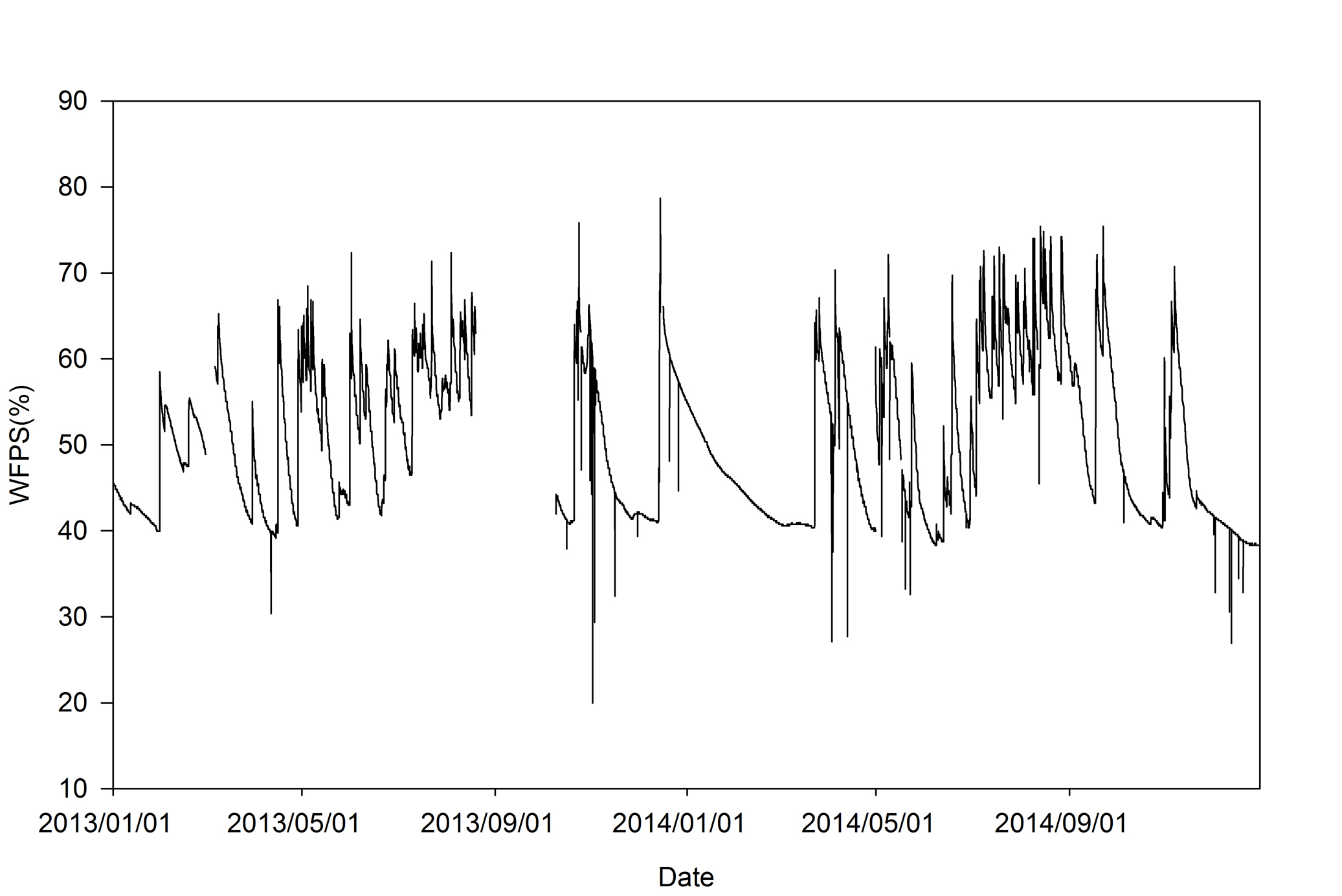
Fig. S2

Table S1 Exponential regressions between the N_2_O flux rate and the soil water content and soil temperature at 5 cm in the rubber plantation in Xishuangbanna, Southwest China

|  | Treatment | a | b | r^2^ | p |
| --- | --- | --- | --- | --- | --- |
|  | NN | 0.0004 | 0.150 | 0.270 | <0.001 |
| T5 | NN+ | 0.00507 | 0.165 | 0.218 | <0.001 |
|  | NNt | 0.00127 | 0.092 | 0.098 | <0.001 |
|  | UN | 0.00110 | 0.131 | 0.230 | <0.001 |
|  | UN+ | 0.00191 | 0.116 | 0.174 | <0.001 |
|  | UNts | 0.540 | 0.0500 |  | 0.386 |
|  | UNta | 0.0006 | 0.347 | 0.326 | <0.001 |
|  | NN | 0.00211 | 5.856 | 0.172 | <0.001 |
| SWC | NN+ | 0.000425 | 13.840 | 0.608 | <0.001 |
|  | NNT | 0.00417 | 2.984 | 0.039 | 0.017 |
|  | UN | 0.00324 | 6.319 | 0.230 | <0.001 |
|  | UN+ | 0.00105 | 10.856 | 0.674 | <0.001 |
|  | UNts | 0.00508 | 11.92 | 0.160 | <0.001 |
|  | UNta | 0.0485 | 3.838 | 0.009 | 0.153 |

Note: the regression is y = ae^(b*x)^, where y is the N_2_O daily flux rate (mg N m^–2^ h^–1^), b is the soil temperature at 5 cm or soil water content.

NN, NN+, and NNt represent the platforms, slopes, and old fertilized trenches of the NF treatment, respectively, and UN, UN+, UNts, and UNta indicate the platforms, slopes, spring-fertilized trench plots, and rainy-season-fertilized trench plots in the F treatments, respectively.

Table S2 Linear regression equations between the soil water content from 0–5 cm, the soil temperature at 5 cm, and the sampling plots and flux tower

|  | | *R^2^* | *p* | *a* | *b* |
| --- | --- | --- | --- | --- | --- |
| Soil water content from 0 to 10 cm depth | NN | 0.2718 | 0.0000 | 14.87 | 0.59 |
|  | NN+ | 0.2682 | 0.0000 | 14.88 | 0.61 |
|  | NNT | 0.2266 | 0.0000 | 17.28 | 0.55 |
|  | UN | 0.2973 | 0.0000 | 11.17 | 0.69 |
|  | UN+ | 0.1938 | 0.0000 | 16.46 | 0.51 |
|  | UNts | 0.2631 | 0.0000 | 12.62 | 0.64 |
|  | UNta | 0.3281 | 0.0000 | 10.56 | 0.71 |
| Soil temperature  at 10 cm depth | NN | 0.9735 | 0.0000 | -1.21 | 1.04 |
|  | NN+ | 0.9696 | 0.0000 | -0.37 | 1.01 |
|  | NNt | 0.9705 | 0.0000 | -1.06 | 1.04 |
|  | UN | 0.7643 | 0.0000 | -5.86 | 1.24 |
|  | UN+ | 0.7229 | 0.0000 | 2.32 | 0.88 |
|  | UNts | 0.9422 | 0.0000 | -1.44 | 1.05 |
|  | UNta | 0.9756 | 0.0000 | -0.84 | 1.02 |

Y = a + bx

where y represents the sampling plot data and x is the flux tower data; and a and b are coefficients.

NN, NN+, and NNt indicate the platforms, slopes, and the old fertilizer trenches of the NF treatment, respectively, and UN, UN+, UNts, and UNta indicate the platforms, slopes, dry-season-fertilized trench plots, and rainy-season-fertilized trench plots in the F treatments, respectively.

**Table S3** Mean N_2_O flux rates (mg N m^–2^ h^–1^) from different treatments in the rubber plantation

| Treatments | Mean (*n*=118) | SE |
| --- | --- | --- |
| NN | 0.0166_a_ | 0.0020 |
| NN+ | 0.0318_a_ | 0.0029 |
| NNt | 0.0140_a_ | 0.0016 |
| UN | 0.0284_a_ | 0.0028 |
| UN+ | 0.0339_a_ | 0.0024 |
| UNts | 0.5485_b_ | 0.0918 |
| UNta | 0.5801_b_ | 0.1119 |

Different letters after the numbers indicate a significant difference at the 0.05 level.

NN, NN+, and NNt indicate the platforms, slopes, and the old fertilizer trenches of the NF treatment, respectively, and UN, UN+, UNts, and UNta indicate the platforms, slopes, dry-season-fertilized trench plots, and rainy-season-fertilized trench plots in the F treatments, respectively.

Table S4 Physicochemical properties of the topsoil layer (0–20 cm) in the rubber plantation

| Parameter | Value |
| --- | --- |
| pH | 5.07 |
| SOM (gC/kg) | 25.09 |
| Total N (g/kg) | 1.38 |
| Total P (g/kg) | 0.38 |
| Ratio T:N | 18.21 |
| Total K (g/kg) | 14.1 |
| Clay fraction (<0.002 mm) % | 33.56 |
| Silt fraction (0.002–0.05 mm) % | 31.90 |
| Sand fraction (0.05–2 mm) % | 34.45 |
|  |  |
